# Variability of mitochondrial DNA D-loop sequences in Zabaikalskaya horse breed

**DOI:** 10.18699/VJ21.055

**Published:** 2021-09

**Authors:** L.A. Khrabrova, N.V. Blohina, B.Z. Bazaron, T.N. Khamiruev

**Affiliations:** All-Russian Research Institute for Horse Breeding, Divovo, Ryazan Region, Russia; All-Russian Research Institute for Horse Breeding, Divovo, Ryazan Region, Russia; Scientif ic Research Institute of Veterinary Medicine of Eastern Siberia – Branch of the Siberian Federal Scientif ic Centre of Agro-BioTechnologies of the Russian Academy of Sciences, Chita, Russia; Scientif ic Research Institute of Veterinary Medicine of Eastern Siberia – Branch of the Siberian Federal Scientif ic Centre of Agro-BioTechnologies of the Russian Academy of Sciences, Chita, Russia

**Keywords:** genetic diversity, haplogroups mtDNA, horse, phylogenetic analysis, Zabaikalskaya breed, генетическое разнообразие, гаплогруппы мтДНК, лошадь, филогенетический анализ, забайкальская порода

## Abstract

The Zabaikalskaya horse is an indigenous breed of horses from Siberia with diverse use. It is characterized by endurance and good adaptability to year-round herd maintenance in the harsh conditions of the Baikal steppes. To determine the genetic characteristics of the maternal lineage of the Zabaikalskaya horse breed based on mitochondrial DNA polymorphisms, we collected hair samples from 31 horses belonging to breeding farms in the Trans-Baikal Territory. Analysis of the 530 bp sequence of the mtDNA D-loop was performed using the maximum composite likelihood (MCL) model in combination with bootstrap analysis. When studying the polymorphism of the hypervariable region of the mtDNA D- loop in Zabaikalskaya horses, we identif ied 31 haplotypes representing 8 haplogroups: B, C, G, H, L, M, Q and R according to modern classif ication. The sequenced fragment of the D-loop from nucleotide position 15471 to 16000 contained 17 polymorphic sites, mainly represented by the A→G, G→A and T→C transitions. The haplogroups Q (25.81 %), B (19.35 %), G (16.13 %) and H (12.90 %) were prevailing in the mtDNA structure of this breed. Genetic analysis of the mitochondrial genome of the Zabaikalskaya horse revealed a high level of diversity of haplotypes and haplogroups, which are typical for the horse populations of Eurasia.

## Abstract (ru)

Забайкальская лошадь – аборигенная порода лошадей Сибири универсального использования. Она характеризуется выносливостью и хорошей приспособленностью к круглогодичному табунному содержанию в суро- вых условиях байкальских степей. Для определения генетических особенностей материнских линий забайкальской породы на основе полиморфизмов митохондриальной ДНК мы собрали образцы волос от 31 лошади, принадлежащей племенным хозяйствам Забайкальского края. Анализ последовательности D-петли мтДНК размером 530 п. н. проводили с использованием модели максимального правдоподобия (MCL) в сочетании с бутстрэп-анализом. При изучении полиморфизма гипервариабельного региона D-петли мтДНК у забайкальских лошадей был выявлен 31 гаплотип, представляющий восемь гаплогрупп: B, C, G, H, L, M, Q и R, согласно современной классификации. Секвени- рованный фрагмент D-петли (нуклеотидная позиция 15471–16000) содержал 17 полиморфных сайтов, в основном представленных транзициями A→G, G→A и T→C. В структуре мтДНК породы преобладали гаплогруппы Q (25.81 %), B (19.35 %), G (16.3 %) и H (12.90 %). Генетический анализ митохондриального генома забайкальской лошади выявил высокий уровень разнообразия гаплотипов и гаплогрупп, которые типичны для популяции лошадей Евразии.

## Introduction

Zabaikalskaya horse is one of the native horse breeds from Siberia and it is distributed in the territories located to the south and southeast of Lake Baikal. The breed was formed through process of long interbreeding of Mongolian horses and later adapted to year-round herd maintenance. The Zabaikalskaya horse is characterized by low growth rate but with a massive and bony appearance. In 1993, these horses were given the status of the Zabaikalskaya breed, and it was included in the State Register of Breeding Achievements of the Russian Federation (Khamiruev et al., 2014).

Today, modern genetics technology has allowed researchers to unravel some of the mysteries surrounding the development of Russian native breeds. The study of features of nuclear and mitochondrial DNA of horses of different breeds and areas, including the found remains of ancient horses, allowed clarifying of many important questions of Equids evolution. In particular, high variability of mitochondrial DNA was revealed, indicating the presence of multiple wild ancestors in domestic horses and the existence of different regions of domestication (Bowling, Ruvinski, 2000; Jansen et al., 2002). Due to the study of the mitochondrial genome, it was finally established that the species *Equus caballus* is not a direct descendant of the wild horse *E. ferus przewalski*.

The study of the mitochondrial genome of the horse began with the work of X. Xu and U. Arnason (1994), which led to the sequencing of equine mtDNA and demonstrated that its configuration varies due to the different number of GTGCACCT repeats in the control region. Further studies have shown that mtDNA polymorphism can be studied using various technologies, among which the method of direct sequencing of the hyper variable region of the D-loop is most often practiced. This approach allows to study matrilineal diversity within horse breeds and their phylogenetic relationships (Bowling et al., 2000; Hill et al., 2002; Lopes et al., 2006; McGahern et al., 2006; Glazewska et al., 2007; Moridi et al., 2012; Vilstrup et al., 2013).

Comparative analysis of mitochondrial DNA in different populations and breeds of horses in Europe and Asia and phylogenetic reconstruction made on its basis showed the presence of complex variability of mitochondrial haplogroups; this was not observed in other domesticated species (McGahern et al., 2006). Comparison of European and Asian horse breeds revealed differentiated distribution of mtDNA haplogroup variants and evidence of a biogeographic wedge in Asian populations, including association of "Eastern" mtDNA types with haplogroup F. A number of previously unknown additional mtDNA sequences were obtained from horses of Akhal-Teke, Vyatskaya, Mezenskaya, Orlov Trotter and Yakutskaya breeds from Russia, among which the greatest similarity with the European populations was observed in the Mezenskaya horse (McGahern et al., 2006).

A new stage of studying the mitochondrial genome of the horse began in 2012, when a team of researchers led by A. Achilli conducted a complete sequencing of 83 mitochondrial genomes of modern horses in Europe, Asia, the Middle East and America. The phylogenetic analysis with high molecular resolution revealed 18 main haplogroups (A–R) with their diagnostic mutational motifs that arose during the Neolithic period. The researchers concluded that the proposed classification of the encoded and control regions of the mitochondrial genome could be used in the study of the remains of ancient horses, phylogenetic relations of modern breeds, intra-breed diversity and evaluation of the possible connection of mtDNA with racing performance.

Subsequent studies of mitochondrial DNA polymorphism in horses of different populations showed that the number of haplogroups in breeds can vary from 4 to 14 (Bigi et al., 2014; Sorokin, 2015; Cardinali et al., 2016; Khrabrova et al., 2020). The study of mitochondrial sequence variation in 251 Arabian horses from different countries showed that they belong to 13 mtDNA haplogroups based on Achilli (Khanshour, Cothran, 2013). The greatest number of mtDNA haplotypes and haplogroups was revealed in Arab horses of the Syrian population, that is, in the region of creation of this breed. Arabian horses had a much wider range of mitochondrial haplotypes compared to Thoroughbred, with a high frequency of haplogroup L in all populations.

When studying the mitochondrial genome of horses, researchers found a clear association of dam lines with certain mtDNA haplogroups and haplotypes (Hill et al., 2002; Lopes et al., 2006; Sorokin, 2015; Khrabrova et al., 2019). This allows, if necessary, to control the female line of horses using the method of mtDNA sequencing (Bowling et al., 2000; Khrabrova et al., 2020).

The task of our research was to study the variability of the D-loop sequence of mtDNA and maternal lineage in the native Zabaikalskaya horse breed, bred in the steppes of Transbaikalia.

## Material and methods

To analyze the 530 bp hypervariable region of mtDNA D-loop we sequenced 31 Zabaikalskaya horses from Chita State Stable (n = 13) and Kalinin stud (n = 18) located in the Trans-Baikal Region. DNA was isolated from hair follicles using a set of ExtraGene DNA Prep 200, produced by laboratory "Isogen" (Moscow), according to the manufacturer's instructions. The original primers for amplification of the studied mtDNA D-loop site were selected by S. Sorokin (2015) taking into account the reference sequence of the fossil Swedish horse X79547 (Xu, Arnason, 1994).

For PCR amplification we used the composition of the reaction mixture included 0.2 mM dNTP, 0.5 μM of each primer, 2.5mM MgCl2, 1× PCR buffer, 1 unit Taq polymerase (PE Applied Biosystems, USA) and 1 unit AmpliTaq Gold polymerase (PE Applied Biosystems), 50 ng DNA. The procedure involved heating the reaction mixture at 95 °С (5 min), followed by 30 cycle's denaturation at 94 °С (30 s), annealing at 55 °С (30 s) and elongation at 72 °С (45 s). Final elongation was carried out at 72 °С for 2 min. The sequencing of the PCR fragment from agarose gel was performed using the Bigdye Terminator Cycle Sequencing Kit (PE Applied Biosystems) on the ABI 3130xl genetic analyzer (PE Applied Biosystems) using manufacturer's protocol.

All mtDNA sequences were prepared with BioEdit 7.2.1. Additional GenBank data set of 18 known haplogroups (A–R) under the number JN398377–JN398457 was used to identify the obtained nucleotide sequences. The phylogenetic analysis of the mtDNA D-loop, including a 530 bp fragment (position 15471–16000) was performed using the Neighbor- Joining (NJ) method and program MEGA 7 (www.megasoftware. net). The statistical confidence of each node was estimated by 1000 random bootstrap runs. When constructing a phylogenetic tree, bootstrap values of more than 50 % were taken into account. For comparison of mtDNA haplogroups distribution in Zabaikalskaya horse, data from 156 Kabardinian, 64 Mongolian (Khaudov et al., 2018), 74 Thoroughbred (Khrabrova et al., 2019) and 22Vyatskaya horses (Khrabrova et al., 2020) were used.

## Results

Sequence analysis of the 530 bp region of the mitochondrial D-loop of Zabaikalskaya horses showed the presence of 31 haplotypes corresponding to 8 haplogroups: B, C, G, H, L, M, Q and R according to Achilli's classification (2012). The sequenced fragment of the D-loop from 15471 to 16000 nucleotide position contained 17 polymorphic sites, mainly represented by the A-G, G-A and T-C transitions (Table 1).

**Table 1.**
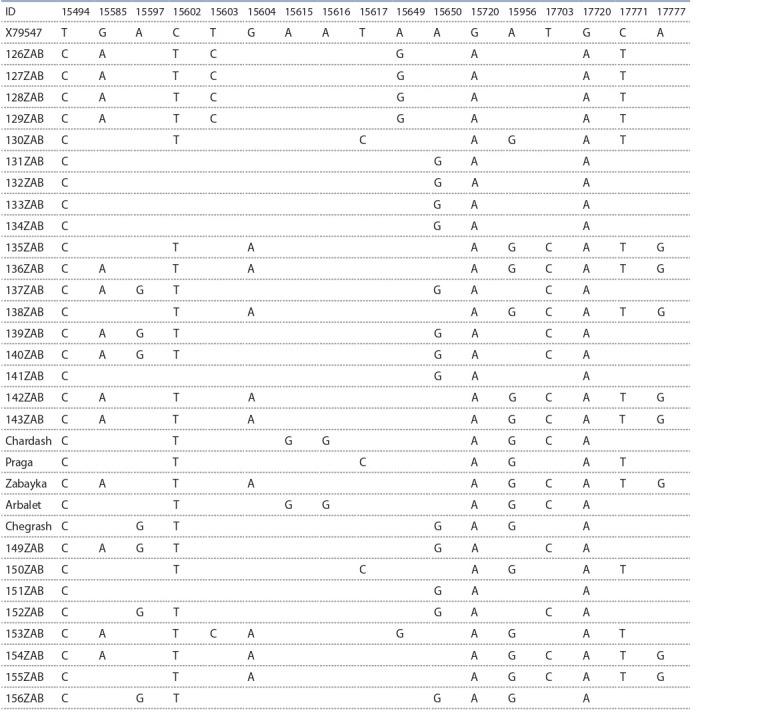
Variability of nucleotides in a 530 bp fragment of the mtDNA D-loop of Zabaikalskaya horse haplotypes compared with reference sequence X79547

The average number of substitutions per site was 0.03± 0.022, indicating a relatively high level of nucleotide diversity.

**Fig. 1.**
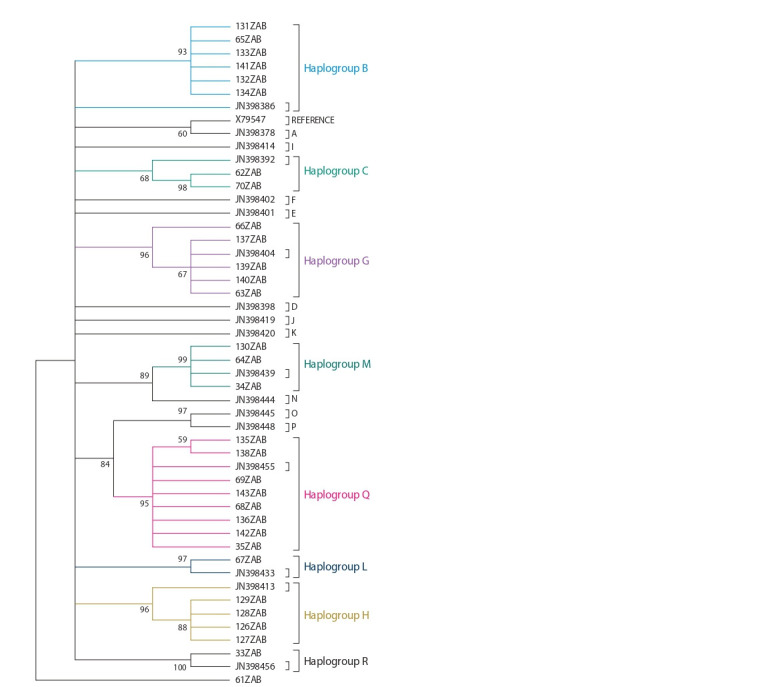
Phylogenetic tree of mtDNA D-loop sequences from haplotypes of Zabaikalskaya horses constructed by use of the Neighbor-Joining method in combination with bootstrap analysis (bootstrap value > 50 are shown at nodes). For identif ication of haplogroups were used GenBank data (JN398377– JN398457) and their classif ication according to (Achilli et al., 2012).

**Fig. 2.**
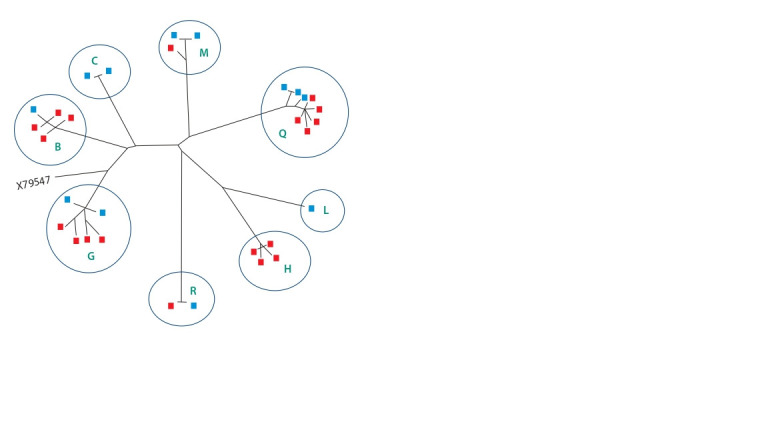
Scheme of evolutionary relationships between Zabaikalskaya horses from different subpopulations (Chita State Stable – blue, Kalinin stud – red) derived from phylogenetic analysis using Neighbor-Joining method and MEGA7 program.

Sequences with haplogroups B, G, M and Q were found in both subpopulations of Zabaikalskaya horses. In addition, mtDNA haplogroups C, L and R were found in horses of the Chita State Stable, while haplogroup H was found only in the Kalinin stud. The genetic structure of Zabaikalskaya horse was dominated by haplogroups Q (25.81 %), B (19.35 %), G (16.3 %) and H (12.90 %), which is clearly demonstrated by the dendrograms in Fig. 1 and 2. Haplotype Q, which combines horses from different farms, is the most common in Asia and the Middle East (Achilli et al., 2012; Khanshour, Cothran, 2013).

The statistical analysis of phylogenetic relationships calculated by the MCL distances shows a fairly high level of bootstrap value (94–100) matching of haplotypes within haplogroups (see Fig. 1). The genetic divergence of the mitochondrial genome of Zabaikalskaya horses from different subpopulations is shown in Figure 2.

Most haplotypes of Zabaikalskaya horse (74.19 %) are found in the haplogroups B, G, H and Q; haplogroup L was rarely found, which is typical of many cultural and local breeds (Table 2).

**Table 2.**
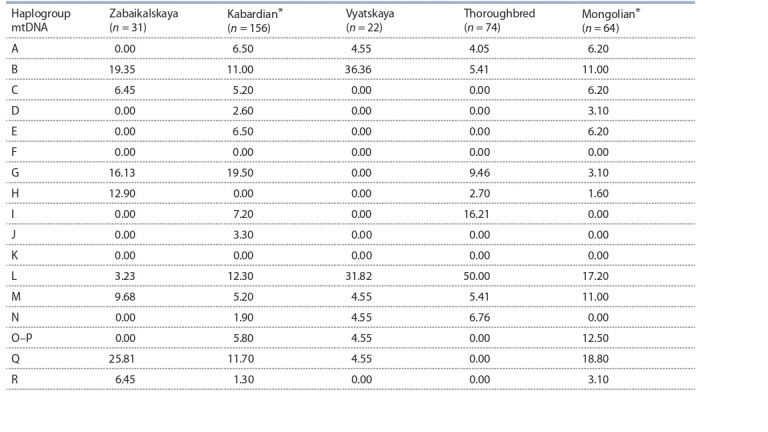
Distribution of the haplogpoups mtDNA (in %) in different horse breeds Date from A.D. Khaudov et al. (2018).

Comparative analysis of mtDNA in horses of different breeds bred in Russia shows that, as a rule, they have a multilinear origin on the maternal line. At the same time, haplogroups B and L were represented quite significantly in all breeds (5.41–36.36 and 3.23–50.0 %, respectively). The genetic analysis of mtDNA of Zabaikalskaya horse breed indicates a high level of diversity of haplotypes and a clear differentiation of I maternal line in the subpopulation of this breed.

## Discussion

The high level of diversity of the genetic structure of mitochondrial DNA and maternal inheritance make it a unique object for the study of evolutionary processes, phylogenetic analysis and evaluation of population diversity (Lukashov, 2009). We found a high mitochondrial polymorphism represented by 31 D-loop haplotypes from 8 haplogroups: B, C, G, H, L, M, Q and R based upon Achilli et al. (2012). In addition, haplogroups A (12.5 %) and D (8.3 %) were identified in the sequences of 24 Zabaikalskaya horse presented in GenBank (Khaudov et al., 2018).

The genetic structure of Zabaikalskaya horse breed shown by mtDNA D-loop haplogroups is generally typical for horse populations in Asia, in which haplogroups B, C, G, and Q were most common (Achilli et al., 2012; Khanshour, Cothran, 2013; Khaudov et al., 2018). It is obvious that the Mongolian horse had a great influence on the formation of Zabaikalskaya horse. The haplogroup variants obtained from the Zabaikalskaya horse were more similar to other local breeds from Siberia (Voronkova, Stolpovskiy, 2018), which indicates their common matrilineal genealogy.

A comparative analysis of polymorphism of microsatellite loci in local horse breeds in Russia showed that Zabaikalskaya horse is characterized by a relatively high level of genetic diversity and has a high level of similarity with Buryat horse (Khrabrova, 2015). Mitochondrial analysis confirms the close relationship of these neighboring breeds, which share 6 common haplogroups: B, G, L, M, Q and R.

Comparison of European and Asian horse breeds revealed differentiated distribution of mtDNA haplogroup variants and evidence of a biogeographic wedge in Asian populations, including association of "Eastern" mtDNA types with haplogroups Q and R. Interestingly, these haplogroups were identified in horses of the Kabardian and Vyatskaya horse breeds bred in the European part of the Russian Federation (Khaudov et al., 2018; Khrabrova et al., 2020), but were absent in the Thoroughbred and Cleveland Bay horses created in England (Khrabrova et al., 2019; Dell et al., 2020).

The study of the sequence of the hypervariable fragment of the mtDNA D-loop makes it possible to assess the interbreed diversity of horses along maternal lines. The data of many researchers confirm the evidence for biogeographic patterning of mtDNA sequences in Eastern horse populations (McGahern et al., 2006; Khanshour, Cothran, 2013; Khaudov et al., 2018).

## Conclusion

The mtDNA analysis of Zabaikalskaya horse identified 31 different haplotypes clustered in 8 haplogroups (B, C, G, H, L, M, Q and R), which indicates the genetic diversity of the maternal ancestry lines in this breed. Zabaikalskaya horses show high frequencies in haplogroups Q, B, G and H; these haplogroups are also the most common in native horse breeds of Siberia and Mongolian horses. The high variability level of the mitochondrial genome in horses of local breeds may determine good adaptive qualities. The obtained data allow us to supply important information about the genetic features of the existing maternal structure of the Zabaikalskaya horse breed.

## Conflict of interest

The authors declare no conflict of interest.
